# Strengthening Effects of Zn Addition on an Ultrahigh Ductility Mg-Gd-Zr Magnesium Alloy

**DOI:** 10.3390/ma11101942

**Published:** 2018-10-11

**Authors:** Yaobo Hu, Chao Zhang, Tianxu Zheng, Fusheng Pan, Aitao Tang

**Affiliations:** 1 College of Materials Science and Engineering, Chongqing University, Chongqing 400044, China; zhcfrank@163.com (C.Z.); tianxu.zheng@cqu.edu.cn (T.Z.); fspan@cqu.edu.cn (F.P.); tat@cqu.edu.cn (A.T.); 2 National Engineering Research Center for Magnesium Alloys, Chongqing 400044, China

**Keywords:** magnesium alloy, Mg-Gd-Zr-Zn, microstructure, texture, mechanical properties

## Abstract

A newly developed Mg-2Gd-0.5Zr-*x*Zn (*x* = 0.5, 1.0, 2.0, 3.0 wt %) alloy system exhibits significant strengthening by doping with Zn. In order to understand the strengthening mechanism, the microstructure, texture evolution, and mechanical properties of ultrahigh ductility Mg-2Gd-0.5Zr alloys with a Zn addition were systematically investigated. The addition of Zn results in the formation of Mg-Gd-Zn intermetallic compounds along grain boundaries, which encourages grain refinement during hot extrusion via the particle stimulated nucleation (PSN) mechanism. Furthermore, during texture sharpening the pole changes from <20$\bar{2}$1> to <01$\bar{1}$0>, which also occurred in the extruded alloys with Zn addition, which is unfavorable for the basal slip and tensile twinning. Mg-2Gd-0.5Zr-3Zn shows well-balanced strength and ductility with a tensile yield strength (YS) and ultimate tensile strength (UTS) of 285 and 314 MPa, accompanied by a high tensile elongation of 24%. They are superior to those of commercial AZ31. The enhanced strength is attributed to grain refinement, precipitation strengthening, and texture sharpening induced by alloying with Zn. The research result is also of great value to the development of low rare-earth, high strength, and high room temperature ductility magnesium alloy.

## 1. Introduction

Magnesium (Mg) alloys are the lightest metallic structural materials, compared to aluminum, titanium, and steel [1]. With the advantages of low density, high specific strength, good damping capacity, and abundant resources, Mg alloys have attracted considerable attention in an automotive industry responding to energy saving and lightweight strategy [2,3,4,5,6,7]. However, the poor room temperature ductility and limited strength of Mg alloys have retarded their wider application severely. In the past decades, a great deal of research has been devoted to improving the room temperature ductility or strength of Mg alloys by alloying, heat treatment, and severe plastic deformation (SPD) [8,9,10,11]. However, it is well known that strength and ductility are mutually exclusive; enhancing one often results in the degradation of the other property. It is therefore difficult to obtain high strength with excellent ductility simultaneously. Thus, developing well-balanced strength and ductility Mg alloys is a critical strategy for further extending the potential usage of Mg alloys.

Alloying addition with rare earth (RE) elements is an effective approach to enhancing strength as well as tailoring texture [12,13,14,15,16,17]. Many studies have been reported that adding the RE element of Gd, Y, Nd into pure Mg can greatly enhance strength through precipitation strengthening and weaken basal texture during hot deformation [18,19]. N. Stanford [20] studied the effects of RE elements on texture modification, and elucidated that RE-containing alloys (such as Mg-Gd and Mg-Y) could improve room temperature ductility due to marked weakened texture. T. Homma and S. Kamado [21] reported that Mg-10Gd-5.6Y-1.6Zn-0.6Zr (wt %) alloy show a high ultimate tensile strength (UTS) of 542 MPa after hot extrusion followed by aging. But the room temperature ductility of wrought Mg alloy is overwhelmingly sacrificed by adding such a large number of RE elements. We are committed to developing a new wrought Mg alloy with low RE content and special texture components—“RE-texture”. Fortunately, an ultrahigh ductility Mg-Gd-Zr alloy was successfully prepared by traditional hot extrusion in our previous research [22]. However, in terms of structural applications it needs to further improve strength.

Zn is generally regarded as a favorable alloying element for enhancing strength by increasing age hardening response, producing intermetallic compounds, and refining grain size [23,24,25]. In the past decade, Mg–RE–Zn alloy systems have attracted substantial attention, due to solution strengthening, aging strengthening, and long period stacking order (LPSO) structures strengthening [26,27,28,29]. However, most of these mainly focus on high Gd concentration, and there are rare studies involved in a dilute Mg-Gd-Zr alloy system.

In this work, we prepared Mg-2Gd-0.5Zr-*x*Zn (*x* = 0.5, 1.0, 2.0, 3.0 wt %) magnesium alloys by traditional hot extrusion. The purpose of the present study is to evaluate the effects of Zn addition on microstructure, texture evolution, and mechanical properties of ultrahigh ductility Mg-2Gd-0.5Zr alloy. Meanwhile, the strengthening mechanism of Zn addition was also studied.

## 2. Materials and Methods

The investigated Mg-2Gd-0.4Zr-xZn alloy ingots were prepared by semi-continuous casting using raw materials of pure Mg (99.98 wt %), pure Zn (99.8 wt %), Mg-20Gd, and Mg-20Zr (wt %) master alloys. The actual chemical compositions of the alloys measured by wavelength dispersive X-ray fluorescence (WDXRF) are listed in Table 1, and hereafter the five Mg-2Gd-0.4Zr-xZn alloys are simply designated as Alloy1, Alloy2, Alloy3, Alloy4, and Alloy5, respectively. Cylindrical ingots with a diameter of 80 mm and 50 mm in gauge length were held at 440 °C for 1 h before the extrusion. Then, the direct extrusion was carried out at 440 °C under the extrusion speed of 40 mm/s with an extrusion ratio of 27. For a comparison of mechanical properties, commercial AZ31 magnesium alloy was selected to be extruded under the same experimental parameters.

The samples for microstructure observation of the studied alloys were cut from an as-cast ingot and as-extruded bar, then polished with SiC emery papers and followed by etching using a mixture of 6 ml of anhydrous ethanol, 1 mL of acetic acid, 1 mL of distilled water, and an appropriate amount of picric acid. Microstructure characterization of these samples was performed using optical microscopy (OM, OLYMPUS, OLS4000) and field emission scanning electron microscopy (FE-SEM, JOEL, JSM-7800F, JEOL. Ltd., Tokyo, Japan) with an energy dispersive X-ray spectrometer (EDS) system. An electron backscattering diffraction (EBSD) test was also conducted using a JSM-7800F FE-SEM equipped with an Oxford Instruments NordlysMax2 EBSD detector, and the accelerate voltage is 20 kV with the scanning step size of 0.5 μm. The samples for the EBSD observation were ground mechanically, followed by electrochemical polishing in a commercial AC2 solution, and the analysis of EBSD data was accomplished by HKL Channel 5.0 software. Transmission electron microscopy (TEM) characterization was operating at 200 kV using a FEI TECNAI G2 F20. The thin foils for the TEM observation were prepared by mechanical grinding to a thickness of approximately 50 µm, then punching into discs with a diameter of 3 mm, and finally argon ion milling using a Gatan 695 precision ion polishing system (PIPS). In addition, the average grain size of as-cast alloys is measured by the linear intercept method from the metallographic image, for as-extruded alloys obtained from EBSD data.

Dog-bone shaped tensile samples with a gauge length of 35 mm and a diameter of 5 mm were tested at room temperature using a universal material machine with an initial strain rate of 2.0 × 10^−3^/s. The tensile direction was parallel to the extrusion direction (ED), and the measurement was repeated at least three times for each alloy to ensure the accuracy of the experiment data.

## 3. Results

### 3.1. Microstructure and Phase Constitution of As-Cast and As-Extruded Alloys

**Figure 1:**
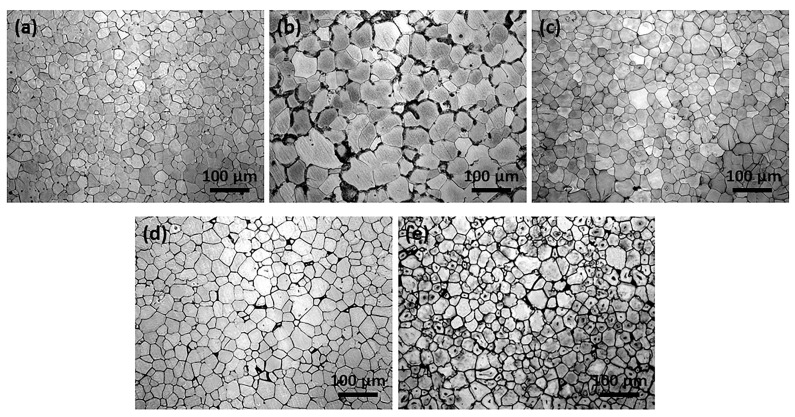
Optical microstructure of the as-cast alloys: (**a**) Alloy1, (**b**) Alloy2, (**c**) Alloy3, (**d**) Alloy4 and (**e**) Alloy5.

The metallographic microstructure of as-cast Mg-2Gd-0.5Zr-xZn alloys (Figure 1) indicate that complete recrystallization occurs on the five alloys during the semi-continuous casting process. The characteristics of equiaxed grain is intrinsically different from that of common AZ31, ZK60, even Mg-Gd and Mg-Zr alloys exhibit a typical configuration of dendritic [30,31,32,33]. That may be closely related to the combined effect of solute atom Gd (dissolved in the matrix) and grain refiner Zr (nucleation sites). The average grain size of as-cast alloys are listed in Table 2; note that Alloy1 has the most uniform and finest grain with an average grain size of 21.6 μm. Unexpectedly, when 0.5 wt % Zn is added, the grain of Alloy2 coarsens evidently (Figure 1b). Reversely, with the further increase of the Zn addition (1.0, 2.0, 3.0 wt %), the grain sizes decreases gradually.

**Figure 2:**
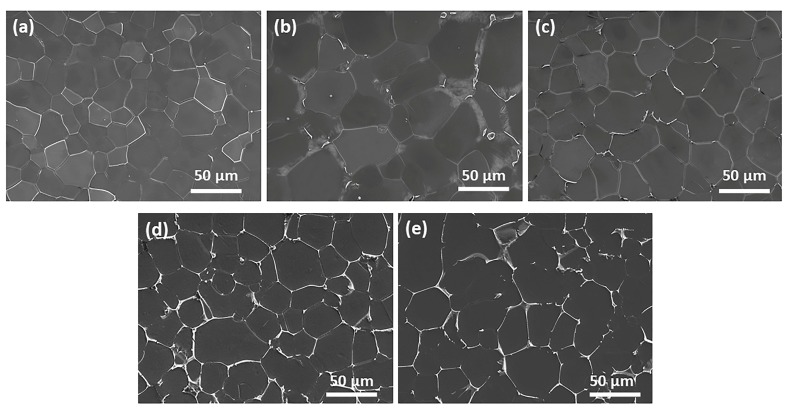
Scanning electron microscope (SEM) images of the as-cast alloys: (**a**) Alloy1, (**b**) Alloy2, (**c**) Alloy3, (**d**) Alloy4 and (**e**) Alloy5.

**Figure 3:**
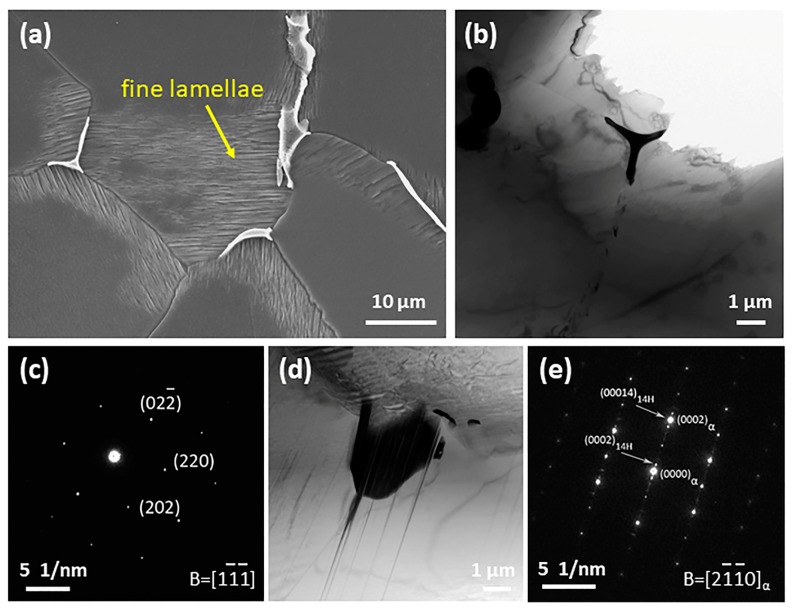
Micrographs of as-cast Alloy2: (**a**) SEM image, (**b**,**d**) Bright field transmission electron microscope (BF TEM) images and (**c**,**e**) their corresponding selected area electron diffraction (SAED) patterns.

**Figure 4:**
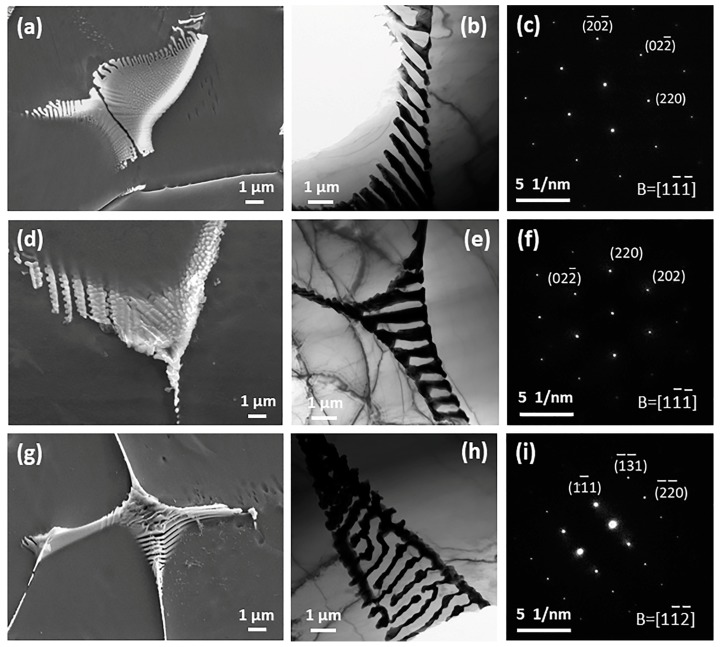
SEM, TEM images and corresponding SAED patterns of as-cast alloys: (**a**–**c**) Alloy3, (**d**–**f**) Alloy4 and (**g**–**i**) Alloy5, respectively.

The SEM images of as-cast Mg-2Gd-0.5Zr-*x*Zn alloys are presented in Figure 2. It is clear that there is no obvious intermetallic compound existing at grain interiors as well as grain boundaries of Alloy1. On the contrary, some irregular shaped intermetallic compounds and intragranular lamellae are observed in Alloy2. The magnified microstructure of Alloy2 (Figure 3a) shows that fine paralleled lamellae are emitted from the grain boundaries to the grain interiors and a small amount of microscale irregular shaped intermetallic compounds grow along the grain boundaries, especially the triple junction region. In order to figure out the phase constitution of Alloy2, bright-field (BF) TEM observation and selected area electron diffraction (SAED) analysis were conducted. The compound was determined to be (Mg,Zn)_3_Gd (with a FCC structure and a lattice constant of a = 0.7158 nm), that is consistent with the findings of A. Srinivasan [34] and J. Zhang [35]. TEM observation shows that the lamellar structure not only locates at the α-Mg matrix, but also forms within the (Mg,Zn)_3_Gd (Figure 3d). Based on the analysis of the SAED pattern (Figure 3e), the lamellar structure was confirmed as 14H type LPSO. Evidently, the fine lamellae with 14H type LPSO structures are formed during solidification, and only appear in a narrow region adjacent to grain boundaries, instead of penetrating the entire grain. With a further increase of Zn, an increasing number of intermetallic compounds were detected at the grain boundaries (Figure 2c–e). It is worth noting that the LPSO structure disappeared totally, instead, while the Zn content exceeds 2 wt %, a network framework composed of abundant intermetallic compounds was formed at the grain boundaries. Figure 4 displays SEM, BF TEM images and corresponding SAED patterns of as-cast Alloy3, Alloy4 and Alloy5, respectively. All of the fishbone-like intermetallic compounds located along the grain boundaries are determined to be W phase (Mg_3_Zn_3_Gd_2_, FCC structure with a lattice constant of a = 0.6857 nm). Similarly, neither (Mg,Zn)_3_Gd nor 14 LPSO structure was observed in as-cast Alloy3, Alloy4 and Alloy5, which may be related to the ratio of Zn/Gd.

**Figure 5:**
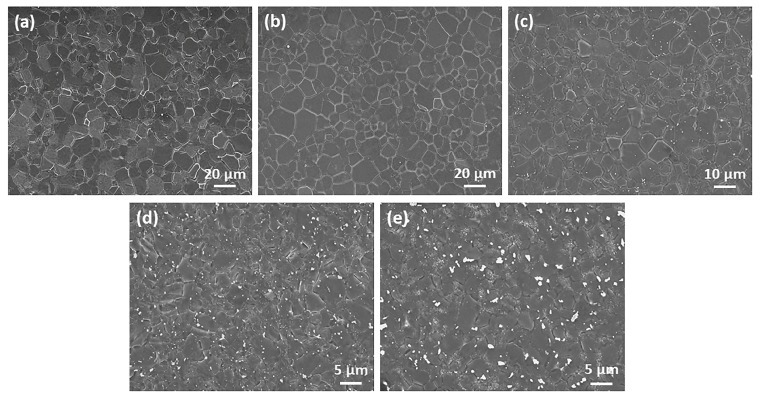
SEM images of as-extruded alloys: (**a**) Alloy1, (**b**) Alloy2, (**c**) Alloy3, (**d**) Alloy4 and (**e**) Alloy5.

**Figure 6:**
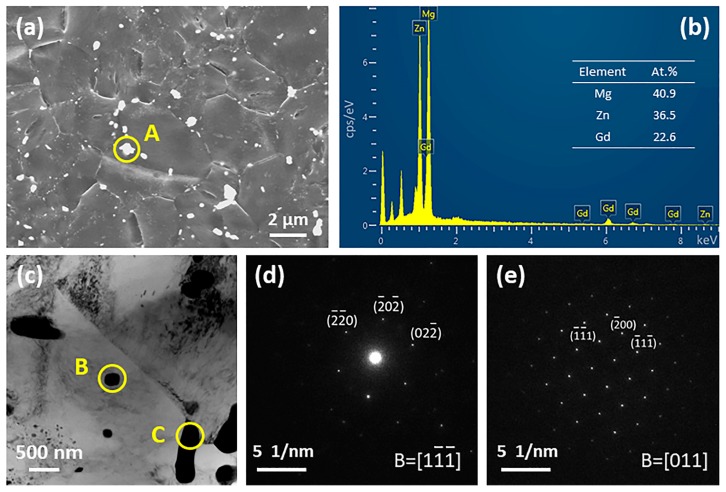
SEM and TEM analysis of as-extruded Alloy4: (**a**,**c**) Micrographs of broken particles, (**b**) energy dispersive X-ray spectrometer (EDS) result of particle A, (**d**,**e**) corresponding SAED patterns of particle B and C, respectively.

The microstructure of the investigated alloys subjected to hot extrusion is exhibited in Figure 5. As revealed, dynamic recrystallization (DRX) occurred during the hot extrusion process, and fine recrystallized equiaxed grains were detected in the five alloys. Furthermore, both Alloy1 and Alloy2 have no significant particle within the clean grain (Figure 5a,b). Interestingly, with respect to (Mg,Zn)_3_Gd and 14 LPSO structure in the as-cast Alloy2, they have disappeared after the hot extrusion process. It is suggested that the alloying element mainly dissolved in the matrix during preheating treatment, instead of dynamic precipitation during the course of hot deformation when the doping amount is insufficient. However, when the Zn content reaches 1.0 wt %, tiny particles appear, with the continuous increase of Zn content, these particles grow up slightly and become more numerous. Looking closely to the SEM and TEM microphotograph (Figure 6) we can notice that some microscale broken intermetallic compounds distributed along the grain boundaries and nanoscale spherical precipitations are located within the grain interiors. Further, EDS and SAED analysis show that the observed microscale and nanoscale particles are W phase (Mg_3_Zn_3_Gd_2_), deriving from the broken fishbone-like intermetallic compounds and dynamic precipitation during hot extrusion, respectively.

### 3.2. Mechanical Properties of As-Extruded Alloys

**Figure 7:**
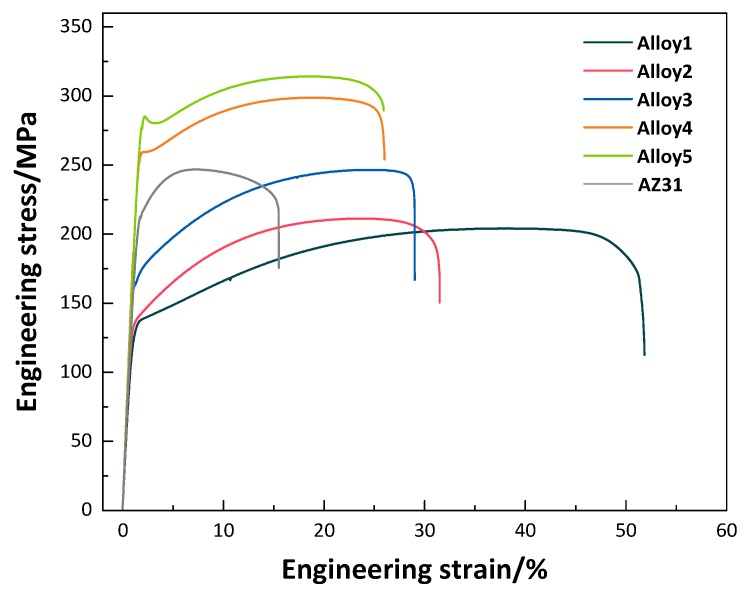
Typical engineering stress-strain curves of the as-extruded alloys.

Typical tensile engineering stress-strain curves of the as-extruded alloys are illustrated in Figure 7. Table 3 summarizes their concrete tensile yield strength (YS), UTS and tensile elongation after fracture (A), respectively. As presented, Alloy1 exhibits the highest room temperature ductility of 51%, approximately three times higher than that of commercial AZ31, which satisfies the level of room temperature ultrahigh ductility (tensile elongation high than 45%). Besides, significant enhancement in YS and UTS of as-extruded alloys are observed with a Zn content increased from 0 to 3.0 wt %, but tensile elongation has dropped. Combined with Table 3, Alloy5 exhibits a larger increase in YS and UTS compared to the Alloy1 and AZ31, yet surprisingly, the YS of Alloy5 is 2.1 times as high as that of Alloy1, 1.4 times of AZ31. Alloy5 shows excellent comprehensive mechanical properties, which has YS and UTS of 285 and 314 MPa, accompanied with A of 24%.

### 3.3. Texture of As-Extruded Alloys

**Figure 8:**
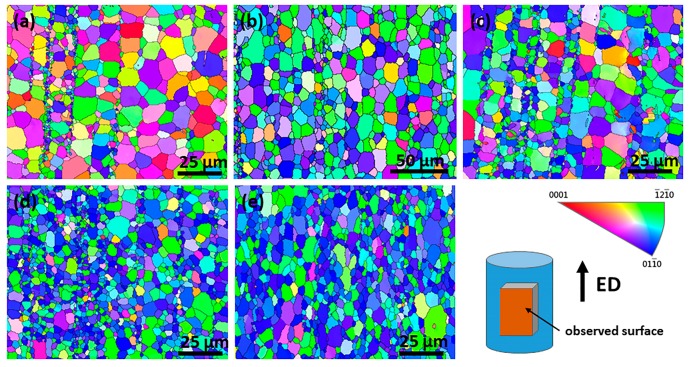
EBSD IPF maps of as-extruded alloys: (**a**) Alloy1, (**b**) Alloy2, (**c**) Alloy3, (**d**) Alloy4 and (**e**) Alloy5.

**Figure 9:**
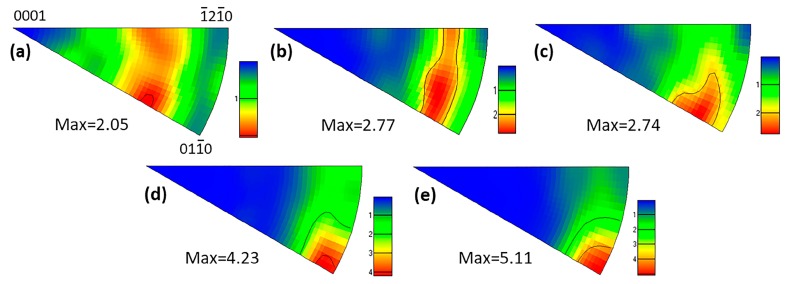
Inverse pole figures of as-extruded alloys refer to ED: (**a**) Alloy1, (**b**) Alloy2, (**c**) Alloy3, (**d**) Alloy4 and (**e**) Alloy5.

Figure 8 displays the EBSD IPF maps of the as-extruded alloys. It shows that the microstructure in the five alloys exhibits similar features characterized by bimodal distribution of grain, uniform equiaxed grain, and dispersive fine grain band. The fine grain band is obviously distinct from extrusion shear band reported by S. Sandlöbes and Z.R. Zeng [36,37,38], which penetrates throughout the the whole extruded bar and parallels each other along ED. Average grain size of extruded Alloy1 is approximately 3.1 μm, unexpectedly, this value of Alloy2 has doubled (6.4 μm). In addition, when the Zn content is up to 1.0 wt %, the average grain size of extruded Alloy3 decreases dramatically, however, with the further increase of the Zn addition, the change of average grain size is not very obvious. Inverse pole figures of as-extruded alloys refer to ED are presented in Figure 9. Alloy1 exhibits a characteristic of RE-texture with a crystallographic orientation of <20$\bar{2}$1> parallel to ED, varying from the typical extrusion fiber texture. Alloying with the Zn element into Mg-2Gd-0.5Zr shifts the texture pole from <20$\bar{2}$1> to <01$\bar{1}$0>, which is a typical basal texture with <01$\bar{1}$0> parallel to ED, consistent with that of AZ31 [39]. What is more, the maximum intensity of pole increases from 2.05 to 5.11 mrd with increasing Zn content.

## 4. Discussion

### 4.1. Effects of Zn Addition on Microstructure

Alloy1 exhibits the characteristics of fine grain structure both in the cast and extruded states. It is reported that Gd has great solubility in pure Mg, even 4 wt % concentrations at 200 °C [19], which totally gets rid of any potential particle stimulated nucleation (PSN) effects during solidification, because PSN occurs when the particle diameter is greater than 1 μm. Consequently, Gd may play an important role in grain boundary migration and grain growth preference. The recent literatures [40,41] have pointed out that Gd is inclined to segregate or form Gd-rich clusters at grain boundaries, which could potentially retard the movement of grain boundaries and impede grain growth by solute drag. This is responsible for the significant grain refinement in Alloy1 during the hot extrusion process. When 0.5 wt % Zn is added to Alloy1, the LPSO structure is formed within grain, which consumes the Gd atom segregated at the grain boundaries, resulting in the weakness of grain boundary pinning effect. That would account for the observation that Alloy2 has the coarsest grain.

**Figure 10:**
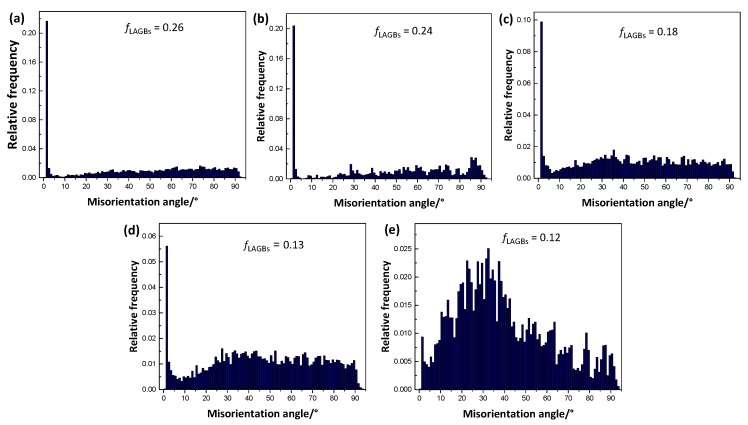
Distribution of grain boundaries misorientation of as-extruded alloys: (**a**) Alloy1, (**b**) Alloy2, (**c**) Alloy3, (**d**) Alloy4 and (**e**) Alloy5.

Figure 10 shows the distribution of grain boundaries misorientation of as-extruded alloys. It is evident that the percentage of low angle grain boundaries (LAGBs) demonstrates a decreasing trend, indicating the LAGBs evolve into high angle grain boundaries (HAGBs) during deformation in Zn-containing alloys. With the increase of Zn content, plenty of Mg-Gd-Zn intermetallic compounds were formed at grain boundaries, and they were broken during the following hot extrusion, and subsequently, promoted the recrystallization by the PSN mechanism. PSN encouraged recrystallization could effectively refine grains, producing an unobvious grain size gradient in Alloy4, 5, as shown in Figure 8. However, the efficiency of grain refinement induced by adding Zn is gradually weakened, considering the PSN caused grain refinement saturates as a dynamic balance between recrystallization refinement and grain growth when the DRX process is complete. As a result, the Alloy3, 4 and 5 have similar average grain size.

### 4.2. Effects of Zn Addition on Texture

Ultrahigh ductility is achieved in diluted Mg-2Gd-0.5Zr at room temperature, which is primarily attributed to RE-texture and fine grain structure induced by hot extrusion. As shown in Figure 2, there are hardly any intermetallic compounds in Alloy1, it can be regarded as a single-phase solid solution, hence, the formation of RE-texture is associated with the segregation of Gd atoms at the grain boundaries. In general, discontinuous DRX (DDRX) would be considered as a dominant softening mechanism accompanied by the characteristic of bulging nucleation at initial HAGBs during thermal deformation. J.P. Hadorn et al. [42] reported that a critical requirement of the DDRX mechanism is HAGBs can be mobile. In this current study, Alloy1 with 2 wt % concentration of Gd has a significant atom segregation at the grain boundaries, which restricts grain boundary mobility and hence suppresses the DDRX process. The DDRX process generally remains the deformation texture rather than replacing it with a recrystallization texture because the orientations of DRX nuclei are different from the orientations of existing grains in the deformation microstructure [42]. Therefore, Alloy1 exhibits a feature of weakened RE-texture distinct with conventional sharp deformation texture of Mg alloys. While adding Zn to Mg-2Gd-0.5Zr based alloys, the broken Mg-Gd-Zn ternary particles provide a basic site for recrystallization nucleation and promotes the DDRX process, resulting in the texture pole changing from <20$\bar{2}$1> to <01$\bar{1}$0> gradually.

### 4.3. Effects of Zn Addition on Deformation Mode

We assess the schmid factor of various slip systems under the loading axis parallels to ED in the five alloys, and the results are displayed in Table 4. As revealed, the maximum value of the schmid factor for basal <a> slip indicates that Alloy1 with RE-texture component of <20$\bar{2}$1> parallel to ED is favorable for the gliding of basal dislocations. With the addition of the Zn element, the texture components change from <20$\bar{2}$1> to <01$\bar{1}$0>, the values of the schmid factor for basal <a> slip decrease gradually, nevertheless, they are opposite for a prismatic <a> slip. It is worth noting that the value of schmid factors for pyramidal <a>, first-order pyramidal <c+a> slip systems remain relatively higher than levels with Zn added to Mg-2Gd-0.5Zr alloys. The changes of Schmid factors reveal that the addition of Zn strengthens basal <a> slip by tailoring the texture from weakened RE-texture to sharp basal fiber texture.

**Figure 11:**
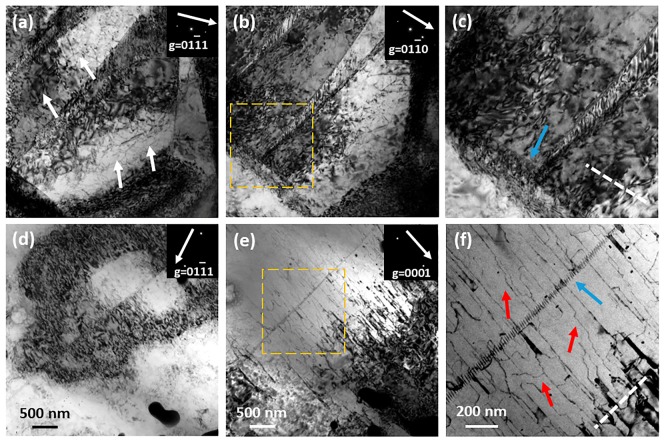
BF TEM images under two-beam condition of as-extruded Alloy1 (**a**–**c**) and Alloy4 (**d**–**f**). The observed samples are subjected to 12% tensile deformation, and the inserts represent diffraction vector in each sample, white dot lines represent basal plane trace.

Bright-field TEM observations of as-extruded Alloy1 and Alloy4 under two-beam condition are illustrated in Figure 11, the samples were subjected to the same tensile deformation (12% engineering strain) along ED at room temperature. According to the g•b criterion (where g is diffraction vector and b is Burgers vectors), dislocations are invisible when g•b = 0 and visible when g•b ≠ 0. It is clear that a high density of dislocations in deformed Alloy1 are visible under g = 01$\bar{1}$1 and g = 01$\bar{1}$0 diffraction conditions (Figure 11a, b), indicating that these dislocations are of <a> type. Since the critical resolved shear stress (CRSS) for prismatic <a> slip is higher than that for the basal <a> slip, it can be confirmed that these are basal <a> type dislocations (denoted by white arrows in Figure 11a). A highly magnified microstructure of the square area in Figure 11b is depicted in Figure 11c, and there is an aligned dislocation wall near the tensile twinning boundary. The dislocation wall indicated by a blue arrow is visible under g = 01$\bar{1}$0 but invisible under g = 01$\bar{1}$1, which is considered a pyramidal <c+a> type dislocation. The frequent activity of basal <a>, pyramidal <c+a> slip, and tensile twinning is responsible for ultrahigh ductility when Alloy1 is subjected to tensile deformation along ED. Some parallel dislocations are observed in deformed Alloy4 under g = 0001 (highlighted by red arrows in Figure 11f), but extinguished from the contrast for g = 01$\bar{1}$1. It can be concluded that these are pyramidal <c+a> type dislocations. Looking closely these dislocations seem to move via a stepped path, J. Jain et al. [43] ovserved that this phenomenon is related to cross-slip of pyramidal <c+a> screw dislocations. They found <c+a> screw dislocations cross-slip from a (11$\bar{2}$2) plane to a (10$\bar{1}$1) plane and then cross-slip back to the original (11$\bar{2}$2) plane again. The pyramidal dislocation wall also appeared in the microstructure of deformed Alloy4. With the addition of Zn, basal <a> slip and tensile twinning are suppressed due to the formation of a sharp basal fiber texture, but partial pyramidal <c+a> slips make a negligible contribution to accommodating plastic strains for Mg-2Gd-0.5Zr-*x*Zn alloys. That may be related to the reduced CRSS for pyramidal slip caused by the solute Gd atom, and the deeper mechanism remains to be further studied.

Based on the analysis above, adding Zn to dilute Mg-2Gd-0.5Zr alloy is an effective approach to improve the YS and UTS simultaneously. There are several factors that account for the enhanced strength. The first is the grain refinement caused by the PSN mechanism. Another is the precipitation strengthening via Mg-Gd-Zn ternary particles impeding the movement of dislocations. Furthermore, in texture sharpening, the pole change from <20$\bar{2}$1> to <01$\bar{1}$0> is unfavorable for the activity of the basal slip and tensile twinning. Although the room temperature tensile elongation decreases with Zn addition, Mg-2Gd-0.5Zr-3.0Zn still exhibits superior ductility than AZ31. This is mainly attributed to the frequent activity of pyramidal <c+a> slips when the extruded samples create tension along the ED.

## 5. Conclusions

The microstructure, texture, and mechanical properties of dilute Mg-2Gd-0.5Zr alloys with variation of Zn addition were investigated by OM, SEM, TEM, and EBSD techniques, in order to understand the strengthening mechanism of the Zn addition. Several conclusions can be drawn, the are as follows:

(1) The concentration of Zn addition has a crucial influence on the phase constitution of Mg-2Gd-0.5Zr-*x*Zn alloys. As Zn content increases from 0.5 to 3.0 wt %, ternary phases change from 14H LPSO + (Mg,Zn)_3_Gd to Mg_3_Zn_3_Gd_2_.
(2) The YS and UTS of the investigated alloys are greatly enhanced with the addition of Zn, which are attributable to grain refinement, precipitation strengthening, and texture sharpening induced by alloying with Zn.
(3) Mg-2Gd-0.5Zr-3Zn exhibits well-balanced strength and ductility with YS and UTS of 285 and 314 MPa, accompanied by a tensile elongation of 24%. The comprehensive mechanical properties are much better than that of AZ31.

## Figures and Tables

**Table 1:** Chemical composition of as-cast alloys.

Designation	Nominal Composition	Actual Composition/wt %			
		Gd	Zr	Zn	Mg
Alloy1	Mg-2Gd-0.5Zr	2.20	0.34	--	Bal.
Alloy2	Mg-2Gd-0.5Zr-0.5Zn	2.13	0.39	0.56	Bal.
Alloy3	Mg-2Gd-0.5Zr-1.0Zn	2.08	0.42	1.10	Bal.
Alloy4	Mg-2Gd-0.5Zr-2.0Zn	1.82	0.50	1.98	Bal.
Alloy5	Mg-2Gd-0.5Zr-3.0Zn	1.78	0.47	2.85	Bal.

**Table 2:** Average grain size of the investigated as-cast and as-extruded alloys.

Condition	Average Grain Size/μm				
	Alloy1	Alloy2	Alloy3	Alloy4	Alloy5
As-cast	21.6	32.8	28.3	26.7	25.4
As-extruded	3.1	6.4	2.8	2.7	2.9

**Table 3:** Tensile properties of the as-extruded alloys at room temperature.

Alloy	YS/MPa	UTS/MPa	A/%
Alloy1	137 (±2)	204 (±1)	51 (±0.4)
Alloy2	142 (±1)	212 (±1)	32 (±0.2)
Alloy3	164 (±3)	246 (±2)	28 (±0.3)
Alloy4	252 (±2)	299 (±1)	25 (±0.2)
Alloy5	285 (±3)	314 (±2)	24 (±0.4)
AZ31	209 (±3)	246 (±4)	14 (±0.3)

**Table 4:** Schmid factors of various slip systems of investigated alloys.

Alloy	Schmid Factor			
	Basal <a> (0001) <11$\bar{2}$0>	Prismatic <a> (10$\bar{1}$0) <1$\bar{2}$10>	Pyramidal <a> (10$\bar{1}$1) <1$\bar{2}$10>	Pyramidal I <c+a> (11$\bar{2}$22) <11$\bar{23}$>
Alloy1	0.37	0.33	0.42	0.43
Alloy2	0.27	0.34	0.43	0.44
Alloy3	0.27	0.39	0.43	0.45
Alloy4	0.21	0.43	0.42	0.45
Alloy5	0.19	0.43	0.42	0.45
